# Supplementary material for: Inflammatory stromal and T cells mediate human bone marrow niche remodeling in clonal hematopoiesis and myelodysplasia
Source: Nat Commun. 2025 Nov 18;16:10042. doi: 10.1038/s41467-025-65803-y (PMC12627676; doi:10.1038/s41467-025-65803-y)
Supplement: Supplementary file 2 — Description of Additional Supplementary Files [file 41467_2025_65803_MOESM2_ESM.pdf]

Description of Additional Supplementary Files

Supplementary Data 1: Donor information, Excel file related to Table 1 and Supplementary Figure 1.
Supplementary Data 2: NanoString Panel analysis, Excel file related to Figure 1 and Supplementary Figure 2.

Supplementary Data 3: Antibodies list used for multimodal approaches (imaging, cell sorting, scRNAseq, FACS diagnostics), Excel file related to Methods and Figure 2 and Supplementary Figure 3.

Supplementary Data 4: scRNA-seq and stromal cell FACS recovery, Excel file related to Figure 2 and Supplementary Figure 4.

Supplementary Data 5: HSPC support, inflammation and angiogenesis gene signatures, Excel file related to Figures 3, 4 and 7.

Supplementary Data 6: Olink and Luminex target panels, Excel file related to Figure 5 and Supplementary Figure 9 and 10.

Supplementary Data 7: Source data to Figures 2B, 2C, 2E, 2G, 3E, 3L, 3M, 5B, 5C, 6B, 6E, 6J, and 7B, and Supplementary Figures 4A-D, 4F-J, and 7D.
