# Supplementary material for: Inflammatory stromal and T cells mediate human bone marrow niche remodeling in clonal hematopoiesis and myelodysplasia
Source: Nat Commun. 2025 Nov 18;16:10042. doi: 10.1038/s41467-025-65803-y (PMC12627676; doi:10.1038/s41467-025-65803-y)
Supplement: Supplementary file 10 — Reporting Summary [file 41467_2025_65803_MOESM10_ESM.pdf]

nature portfolio

Corresponding author(s): Borhane Guezguez, Judith Zaugg

Last updated by author(s): Jul 23, 2025

Reporting Summary

Nature Portfolio wishes to improve the reproducibility of the work that we publish. This form provides structure for consistency and transparency in reporting. For further information on Nature Portfolio policies, see our Editorial Policies and the Editorial Policy Checklist.

Statistics

For all statistical analyses, confirm that the following items are present in the figure legend, table legend, main text, or Methods section.

n/a	Confirmed	
☐	☒	The exact sample size (*n*) for each experimental group/condition, given as a discrete number and unit of measurement
☐	☒	A statement on whether measurements were taken from distinct samples or whether the same sample was measured repeatedly
☐	☒	The statistical test(s) used AND whether they are one- or two-sided<br>*Only common tests should be described solely by name; describe more complex techniques in the Methods section.*
☐	☒	A description of all covariates tested
☐	☒	A description of any assumptions or corrections, such as tests of normality and adjustment for multiple comparisons
☐	☒	A full description of the statistical parameters including central tendency (e.g. means) or other basic estimates (e.g. regression coefficient) AND variation (e.g. standard deviation) or associated estimates of uncertainty (e.g. confidence intervals)
☐	☒	For null hypothesis testing, the test statistic (e.g. *F*, *t*, *r*) with confidence intervals, effect sizes, degrees of freedom and *P* value noted<br>*Give P values as exact values whenever suitable.*
☒	☐	For Bayesian analysis, information on the choice of priors and Markov chain Monte Carlo settings
☒	☐	For hierarchical and complex designs, identification of the appropriate level for tests and full reporting of outcomes
☐	☒	Estimates of effect sizes (e.g. Cohen's *d*, Pearson's *r*), indicating how they were calculated

Our web collection on statistics for biologists contains articles on many of the points above.

Software and code

Policy information about availability of computer code

Data collection	1. Flow cytometry was performed on BD Symphony A3, BD Canto II, and BD FACSAria Fusion using FACSDiva v6 software (BD Biosciences, USA).<br>2. Brightfield imaging of histopathological stains (Giemsa) and immunofluorescence imaging were performed using EVOS M5000 (ThermoFisher) and Opera Phenix (PerkinElmer) respectively, with image acquisition Harmony 4.8 (PerkinElmer).<br>3. scRNA-seq libraries were prepared using the 10x Genomics Chromium and the 10x Genomics NEXT-GEM Single Cell 3' v3.1 kit and sequenced on an Illumina NextSeq2000 and NovaSeqX plus platforms.<br>4. SNP genotyping was performed on isolated gDNA and with the Infinium CoreExome-24 v1.4 BeadChip (Illumina).<br>5. NanoString gene expression profiling was performed using the NanoString nCounter system with the Immune Exhaustion and PanCancer Immune Profiling panels, following the manufacturer's protocol (NanoString Technologies).<br>6. Olink secretome profiling was carried out using a custom Olink® Flex 30 panel and analyzed using real-time PCR (BioMark, Fluidigm) at the DKFZ Olink Core Facility.<br>7. Luminex cytokine profiling was performed using a custom 20-plex ProcartaPlex kit and analyzed using MAGPIX (Uminex Corp, USA).
Data analysis	1. Flow cytometry data were analyzed using FlowJo v10. Statistical testing and graphing were performed in GraphPad Prism v10.4.1.<br>2. Histopathology images were processed using QuPath v0.5 with the MarrowQuant plugin. Statistical testing and graphing were performed in GraphPad Prism v10.4.1.<br>3. Immunofluorescence imaging was analyzed with Harmony 4.8 software (PerkinElmer) and Fiji (ImageJ). Statistical testing and graphing were performed in GraphPad Prism v10.4.1.<br>4. SNP genotyping data was analyzed using the Illumina Array Analysis Platform Genotyping Command Line Interface v1.1, GTCtoVCF v1.2.1, bcftools v1.16 and picard v2.27.4.<br>5. scRNA-seq 10x data from primary patient samples were processed with Cell Ranger v7.0.0 and Souporcell v2.0 and analyzed in R v4.2.2

using Seurat v4.1.1, SoupX v1.6.2, scDblFinder v1.12.0, pySCENIC v0.11.1, AUCell v1.20.2, Monocle v1.3.1, DESeq2 v1.36.0, ashr v2.2.63, BoneMarrowMap v0.1.0, SpliceUp v0.15.2 and clusterProfiler v4.10.1. scRNA-seq 10x data from HSCPC-MSC co-cultures was preprocessed using Cell Ranger v9.0.0 and analyzed using Seurat v4.1.1. CEL-seq2 data was preprocessed using Snakemake v7.32.4, STAR v2.7.10a, and dropEst v0.8.6 and analyzed in R v4.2.2 using Seurat v4.1.1.
6. NanoString data were preprocessed using nSolver software package and nSolver Advanced Analysis module (NanoString Technologies) and analyzed in R v4.2.2 using limma v3.58.1 and clusterProfiler v4.10.1.
7. Olink data were processed using the NPX Manager (Olink Proteomics AB). Normalized protein values (NPX) were further analyzed and graphed in GraphPad Prism v10.4.1.
8. Luminex data were analyzed using MAGPIX software (Luminex Corp, USA). Calculated cytokine concentrations were further analyzed and graphed in GraphPad Prism v10.4.1.

Code used to analyze the data is available at https://git.embl.org/grp-zaugg/BM_CHIP_MDS

For manuscripts utilizing custom algorithms or software that are central to the research but not yet described in published literature, software must be made available to editors and reviewers. We strongly encourage code deposition in a community repository (e.g. GitHub). See the Nature Portfolio guidelines for submitting code & software for further information.

Data

Policy information about availability of data

All manuscripts must include a data availability statement. This statement should provide the following information, where applicable:

- Accession codes, unique identifiers, or web links for publicly available datasets
- A description of any restrictions on data availability
- For clinical datasets or third party data, please ensure that the statement adheres to our policy

Data availability:
Raw count data for single-cell profiling (10x Genomics and CEL-Seq2) and NanoString nCounter gene expression data have been deposited at Figureshare: 10.6084/m9.figshare.27643503.
Raw data are available on the Gene Expression Omnibus (GEO): 10x and CEL-Seq2 (GSE309534, GSE309536), and NanoString (GSE309538, GSE309540). Data not deposited on GEO are available upon reasonable request through the corresponding authors.
For interactive exploration of our scRNA-seq data, we created a Shiny web application: https://shiny-portal.embl.de/shinyapps/app/11_cellmds. The Shiny app was created using the ShinyCell R package 180.
Code availability:
Code used for the analyses of NanoString nCounter gene expression data and single-cell transcriptomic profiling in this paper is publicly available at https://git.embl.de/grp-zaugg/BM_CHIP_MDS.

Research involving human participants, their data, or biological material

Policy information about studies with human participants or human data. See also policy information about sex, gender (identity/presentation), and sexual orientation and race, ethnicity and racism.

Reporting on sex and gender	The biological sex of all human participants is reported in Table 1, Supplementary Table 1, and Figure 1. The cohort included: 27 female and 8 male healthy participants, 10 female and 7 male CHIP participants, and 15 female and 17 male MDS donors. Gender identity was not recorded, as biological sex was the relevant variable.<br>For single-cell analyses, cohort composition was balanced by sex, and potential bias due to sex chromosome–specific gene expression was assessed. No clustering effects by sex were observed in PCA space or in age correlation analyses. In bone marrow cellularity studies (Figure 3B,C; Supplementary Figure 5A–D), no sex bias was detected, including in adipocyte counts.
Reporting on race, ethnicity, or other socially relevant groupings	Data on race, ethnicity, or other socially relevant groupings were not collected for this donor cohort, and no related analyses were performed.
Population characteristics	Additional details on participant age, genotype, and treatment status are provided in Table 1 and Supplementary Table 1. The three groups (healthy controls, CHIP, MDS) were age-matched, with median ages of 71, 77, and 72 years, respectively (range: 60–89 years). CHIP participants mainly carried mutations in epigenetic regulators (DNMT3A, TET2, ASXL1), while MDS participants had SF3B1 or other splicing factor mutations. MDS donors were mainly low-risk and either newly diagnosed or treatment-naïve (no erythropoietin or supportive care).
Recruitment	Human bone marrow aspirates and femur/hip core biopsies were obtained from consenting donors enrolled in the BoHemE study (NCT02867085). Donors and patients were recruited during routine clinical checkups or hip replacement surgery.
Ethics oversight	The use of MDS, CHIP, and control bone marrow samples was approved by local ethics committees at he University Hospital Dresden (TU Dresden, reference nr.: EK 393092016) and University Hospital Leipzig (University of Leipzig, Faculty of Medicine, reference nr.: 137/19-1k) and conducted in accordance with the Declaration of Helsinki.

Note that full information on the approval of the study protocol must also be provided in the manuscript.

Field-specific reporting

Please select the one below that is the best fit for your research. If you are not sure, read the appropriate sections before making your selection.

☒ Life sciences ☐ Behavioural & social sciences ☐ Ecological, evolutionary & environmental sciences

For a reference copy of the document with all sections, see nature.com/documents/nr-reporting-summary-flat.pdf

Life sciences study design

All studies must disclose on these points even when the disclosure is negative.

Sample size	Sample sizes were determined based on prior knowledge and established standards for generating reliable and interpretable data, while ensuring technical and biological feasibility, particularly for sequencing-based experiments. We aimed for a minimum of n=3 for both human and mouse experiments per experimental group, which we deemed sufficient based on the expected effect size. All sample sizes used are reported in the Methods and figure legends. No formal statistical power calculation was performed.
Data exclusions	No data points were excluded. In the single-cell transcriptomics data, we removed cells if they failed pre-established quality control criteria (.e.g., genes per cell, UMIs per cell, mitochondrial content per cell) and excluded clusters that were collectively high in mitochondrial content.
Replication	All experiments were performed with at least three independent biological replicates. Sample sizes for each experiment are mentioned in the figure legends and complementary supplementary tables. Statistical analysis and p/q values are reported for the experiments to assess reproducibility and significance. For in vitro co-culture experiments, each biological replicate consisted of 3 technical replicates (n=3) and the mean was calculated.
Randomization	In in vitro assays, biological replicates were processed in randomized order. In in vivo experiments, animals were randomly assigned to treatment groups to minimize bias. For single-cell experiments, we processed 1 donor / each condition / experimental day, to prevent experimental batch effects.
Blinding	For most in vitro experiments, except the T cell flow cytometry, investigators were not blinded to group allocation due to fixed group design. For in vivo mouse experiments, investigators were blinded during animal allocation, data collection, and analysis. For pathological evaluation of human bone marrow sections, Giemsa stainings and subsequent analyses were conducted in a blinded manner.

Reporting for specific materials, systems and methods

We require information from authors about some types of materials, experimental systems and methods used in many studies. Here, indicate whether each material, system or method listed is relevant to your study. If you are not sure if a list item applies to your research, read the appropriate section before selecting a response.

Materials & experimental systems

n/a	Involved in the study	
☐	☒	Antibodies
☐	☒	Eukaryotic cell lines
☒	☐	Palaeontology and archaeology
☐	☒	Animals and other organisms
☒	☐	Clinical data
☒	☐	Dual use research of concern
☒	☐	Plants

Methods

n/a	Involved in the study	
☒	☐	ChIP-seq
☐	☒	Flow cytometry
☒	☐	MRI-based neuroimaging

Antibodies

Antibodies used

All antibodies used in this study are listed in Supplementary Table 3. Below is the complete list.

Flow cytometry antibodies:
CD2 (FITC, DAKO, clone MT910, cat# DAK-F076701), CD3 (BV421, BD Bioscience, clone UCHT1, cat# 562426; FITC, BD Bioscience, clone SK7, cat# 345764; APC-H7, BD Bioscience, clone SK7, cat# 560176; BV650, BD Bioscience, clone SP34-2, cat# 563916), CD4 (BUV496, BD Bioscience, clone SK3, cat# 612936), CD5 (APC, BD Bioscience, clone L17F12, cat# 345783), CD7 (FITC, BD Bioscience, clone 4H9, cat# 332773), CD8 (BUV395, BD Bioscience, clone RPA-T8, cat# 563795), CD10 (PE-Cy7, BD Bioscience, clone HI10a, cat# 341112), CD11b (PacBlue, Biolegend, clone ICRF44, cat# 301315), CD13 (PE, BD Bioscience, clone L138, cat# 347406), CD14 (FITC, BD Bioscience, clone M5E2, cat# 345784; PE, Biolegend, clone HCD14, cat# 325605), CD15 (APC, Biolegend, clone HI98, cat# 301908), CD16 (FITC, Beckman Coulter, clone 3G8, cat# B49215), CD19 (V450, BD Bioscience, clone HIB19, cat# 560353), CD25 (RY610, BD Bioscience, clone M-A251, cat# 758585), CD26 (KB520, Biolegend, clone BA5b, cat# 302725), CD33 (APC, BD Bioscience, clone P67.6, cat# 345800), CD34 (PerCP-5.5, BD Bioscience, clone 8G12, cat# 347222; PE-Vio770, Miltenyi, clone REA1164/AC136, cat# 130-124-461), CD36 (PE, BD Bioscience, clone CB38, cat# 555455), CD38 (PE, BD Bioscience, clone HB-7, cat# 345806; AF700, BD Bioscience, clone HIT2, cat# 560676), CD44 (FITC, Biolegend, clone QA19A43, cat# 163605), CD45 (V500, BD Bioscience, clone HI30, cat# 560777; BUV805, BD Bioscience, clone HI30, cat# 612892; BV711, BD Bioscience, clone HI30, cat# 564357), CD51/61 (PE-

	Vio770, Miltenyi, clone 23C6, cat# 130-103-746), CD56 (PE-Cy7, BD Bioscience, clone NCAM16.2, cat# 335826), CD64 (PE-Cy7, Biolegend, clone 10.1, cat# 305022), CD71 (AF700, Biolegend, clone CY1G4, cat# 334129; PE, Invitrogen, clone OKT9, cat# 12-0719-42), CD73 (Pacific Blue, Biolegend, clone AD2, cat# 344011), CD90 (BV510, Biolegend, clone 5E10, cat# 328125), CD105 (V450, BD Bioscience, clone 266, cat# 561447), CD117 (APC, BD Bioscience, clone 104D2, cat# 333233; PE-Cy7, BD Bioscience, clone 104D2, cat# 339217), CD123 (PE-Cy7, Biolegend, clone 6H6, cat# 306010), CD140a (APC-Vio770, Biolegend, clone APA5, cat# 135907), CD235a (FITC, eBioscience, clone 10F7MN, cat# 11-9886-42; AF700, Biolegend, clone HI264, cat# 349121; PE, BD Bioscience, clone GA-R2/HIR2, cat# 561051), CD271 (APC, Biolegend, clone ME20.4, cat# 345107; PE-Vio615, Miltenyi, clone REA844, cat# 130112787), CD295 (APC, Miltenyi, clone REA361, cat# 130-124-901), HLA-DR (APC-H7, BD Bioscience, clone L243, cat# 641411), FoxP3 (PE, BD Bioscience, clone 259D/C7, cat# 560046), IL1R1 (PE, R&D Systems, polyclonal, cat# FAB269P), Fixable Viability Dye (FV780, Thermo Fisher, cat# 65-0865-14), Zombie Aqua (Biolegend, cat# 423101).<br><br>Primary antibodies for immunofluorescence microscopy:<br>CD105 (goat polyclonal, R&D Systems, cat# AF1320), CD271 (rabbit monoclonal, Abcam, clone EP1039Y, cat# Ab52987), CD3 (mouse monoclonal, LIFE Technologies, clone F7.2.38, cat# MA5-12577; rabbit polyclonal, Dako, cat# A0452), CD34 (sheep polyclonal, R&D Systems, cat# AF7227; mouse monoclonal, Dako, clone QBEnd10, cat# M716529-2), CXCL12 (mouse monoclonal, R&D Systems, clone 79018, cat# MAB350), FoxP3 (rat monoclonal, Thermo Fisher, clone PCH101, cat# 14-4776-82), GNLY (goat polyclonal, Biotechne, cat# AF3138), HSP60 (rabbit polyclonal, Proteintech, cat# 15282-1), IL1R1 (mouse monoclonal, Thermo Fisher, clone IL1 31-104.1.1, cat# MA1-10858), OASL (rabbit polyclonal, Sigma-Aldrich, cat# HPA001474), UEA1 (FITC-conjugated, VectorLabs, cat# VEC-FL-1061-2; DyLight 649-conjugated, VectorLabs, cat# VEC-DL-1068), BCAM (goat polyclonal, R&D Systems, cat# AF2899), Endomucin (rat monoclonal, Santa Cruz, clone V.7C7, cat# Sc-65495), PDGFR-beta (rabbit monoclonal, Cell Signaling Technology, clone 28E1, cat# 3169S).<br><br>Secondary antibodies for immunofluorescence microscopy:<br>Donkey anti-mouse IgG Alexa Fluor 488 (Thermo Fisher, polyclonal, cat# A21202), Alexa Fluor 568 (cat# A10037), Alexa Fluor 647 (cat# A32787); donkey anti-rabbit IgG Alexa Fluor 488 (cat# A32790), Alexa Fluor 568 (cat# A10042), Alexa Fluor 647 (cat# A31573); donkey anti-goat IgG Alexa Fluor 568 (cat# A11057), Alexa Fluor 647 (cat# A21447); donkey anti-sheep IgG Alexa Fluor 568 (cat# A21099); donkey anti-rat IgG Alexa Fluor 568 (cat# A78946), Alexa Fluor 647 (cat# A48272).
Validation	Antibodies were purchased from the above stated suppliers. All antibodies were validated by the respected companies (also communicated on their website; available with clone and/or catalogue number) and published before. Before usage, we titrated and tested antibodies to find the optimal concentration for flow cytometry or immunofluorescence imaging.

Eukaryotic cell lines

Policy information about cell lines and Sex and Gender in Research

Cell line source(s)	- Human BM stromal cell line hTERT-MSC: were purchased from Applied Biological Materials Inc.(Cat. No. T0523, lot: HC1112) and provided originally by Dr. Charles Mullighan (St. Jude Children's Research Hospital, USA). Donor age and sex was not disclosed, but all relevant information can be found in Mihara, K. et al., Br J Haematol. 2003. (doi: 10.1046/j.1365-2141.2003.04217.x. PMID: 12614220).<br>- MDS-L cell line: provided by Dr. Kaoru Tohyama (Kawasaki Medical School, Japan). Donor was 52 years old, male, and all other relevant information can be found at https://www.cellosaurus.org/CVCL_A8QV.<br>- Human primary BM MSCs: originally isolated from a healthy donor, 32 years old male, and used at passage 1 (P1), after informed consent and in accordance with procedures approved by the local ethics committee (Comité de Protection des Personnes–Île-de-France V, Hôpital Saint-Antoine, Paris) and with the Declaration of Helsinki.
Authentication	All cell lines were authenticated using the Multiplex cellline authentication test by Multiplexion (Heidelberg, Germany).
Mycoplasma contamination	All cell lines and cultures were tested for mycoplasma contamination on a regular basis.
Commonly misidentified lines<br>(See ICLAC register)	N/A

Animals and other research organisms

Policy information about studies involving animals; ARRIVE guidelines recommended for reporting animal research, and Sex and Gender in Research

Laboratory animals	Dnmt3afl-R878H/+ mice (JAX:032289) and B6.Cg-Tg(Mx1-cre)1Cgn/J mice (JAX:003556, referred to as Mx-Cre) were obtained from The Jackson Laboratory, USA
Wild animals	N/A
Reporting on sex	Sex did not play a role in this study. Male and female mice from 6-7 months ("young") and from 15-16 months ("old") were used in all experiments.
Field-collected samples	N/A
Ethics oversight	All animal experiments were reviewed and approved by the The Jackson Laboratory Institutional Animal Care and Use Committee.

Note that full information on the approval of the study protocol must also be provided in the manuscript.

Plants

Seed stocks

N/A

Novel plant genotypes

N/A

Authentication

N/A

Flow Cytometry

Plots

Confirm that:

☒ The axis labels state the marker and fluorochrome used (e.g. CD4-FITC).

☒ The axis scales are clearly visible. Include numbers along axes only for bottom left plot of group (a 'group' is an analysis of identical markers).

☒ All plots are contour plots with outliers or pseudocolor plots.

☒ A numerical value for number of cells or percentage (with statistics) is provided.

Methodology

Sample preparation

Sample preparation of the individual flow cytometry experiments are described in detail in the Methods part of the manuscript.

Instrument

1. Diagnostic immunoprofiling: Canto II (BD Biosciences)
2. T cell subset characterization: Symphony A3 (BD Biosciences)
3. Assessment of stromal surface marker expression in primary BM samples: FACSAria Fusion (BD Biosciences)
4. Cell sorting for scRNA-seq (10x Genomics and CEL-seq2): FACSAria Fusion (BD Biosciences)
5. iMSCs flow cytometry characterization: FACSAria Fusion (BD Biosciences)

Software

FlowJo v10.8 and FACSDiva v6 (BD Biosciences)

Cell population abundance

1. Diagnostic immunoprofiling:
A hierarchical gating strategy based on the iMDSFlow pipeline was applied as follows: (A) exclusion of doublets (FSC-A vs. FSC-H), (B) removal of debris (FSC vs. SSC), and (C) gating of CD45^dim/+ leukocytes (SSC vs. CD45). Within the gated CD45^{+} population, the relative proportions of hematopoietic stem and progenitor cells (HSPCs)/myeloblasts (CD34^{+} or CD117^{+}), CD19^{+} B cells, CD10^{+}/CD19^{+} B cell progenitors, CD3+ T cells, CD14+ monocytes, NK cells (CD56+/CD16+/dim), NKT cells (CD3 +/CD56+), and granulocytes were quantified.
2. T cell subset characterization:
A hierarchical gating strategy was applied as follows: (A) exclusion of doublets (FSC-A vs. FSC-H), (B) removal of debris (FSC vs. SSC), (C) gating of live CD45^{+} hematopoietic cells, followed by identification of CD3^{+} T cells, which were further separated into CD4^{+} and CD8^{+} subsets. Regulatory T cells (Tregs) were defined as CD4^{+}/FoxP3^{+}/CD25^{+} cells. The relative proportions of each T cell subset were quantified.
3. Assessment of stromal surface marker expression in primary BM samples:
A hierarchical gating strategy was applied as follows: (A) exclusion of doublets (FSC-A vs. FSC-H), (B) removal of debris (FSC vs. SSC), (C) selection of live, lineage-negative cells based on exclusion of CD14^{+}, CD71^{+}, and CD235a^{+} populations (Live/Dead vs. lineage markers), (D) gating of CD45low non-hematopoietic cells, and (E) identification of stromal bone marrow cells as CD271^{+}/CD38^{-}. Within the CD45low/CD34^{+}/CD14-/CD71-/CD235a/CD38- population, the relative abundance of putative mesenchymal stromal cells (MSCs) was assessed by co-expression of additional stromal surface markers: CD26, CD73, CD90, CD140a, CD271, and CD295.
4. Cell sorting for scRNA-seq (10x Genomics and CEL-Seq2):
Using the gating strategy described in (3), cells were sorted for single-cell RNA sequencing. For 10x Genomics, the following populations were isolated: HSPCs (CD45mid/CD34^{+}), T cells (CD45^{+}/CD3+), and MSCs (CD45^{-}/CD71^{-}/CD235a^{-}/CD14^{-}/CD38^{-}). For CEL-Seq2, MSCs were further enriched by sorting CD45^{-}/CD14^{-}/CD71^{-}/CD235a^{-}/CD38^{-}/CD271^{+} cells. The purity of post-sort fractions was validated by clustering analysis of cell type–specific gene expression profiles during single-cell transcriptomic analysis, confirming the identity and enrichment of the expected populations.
5. iMSC flow cytometry assessment:
A hierarchical gating strategy consistent with stromal cell profiling (see point 3) was applied. Live, lineage-negative (CD14^{-}/CD71^{-}/CD235a^{-}) non-hematopoietic cells were gated as CD45low, and stromal cells were identified as CD271^{+}/CD38^{-}. Within the CD271^{+} population, mesenchymal stromal cells (MSCs) were further defined by CD73 co-expression. Inflammatory MSCs (iMSCs) were identified as a subpopulation expressing CD44 within the Lin^{-}CD271^{+}CD73^{+} gate. The relative abundance of iMSCs was quantified within this compartment. Also the expression of CD51/61 was assessed.

Gating strategy

All gating strategies are provided in the manuscript information file, in the Methods and in Supplementary Figure 3 (assessment of stromal surface markers and cell gating for scRNA-seq) and Supplementary Figure 6 (iMSC assessment).

☒ Tick this box to confirm that a figure exemplifying the gating strategy is provided in the Supplementary Information.
