# Supplementary material for: Inflammatory stromal and T cells mediate human bone marrow niche remodeling in clonal hematopoiesis and myelodysplasia
Source: Nat Commun. 2025 Nov 18;16:10042. doi: 10.1038/s41467-025-65803-y (PMC12627676; doi:10.1038/s41467-025-65803-y)
Supplement: Supplementary file 1 — Supplementary Information [file 41467_2025_65803_MOESM1_ESM.pdf]

Supplementary Information

Inflammatory stromal and T cells mediate human bone marrow niche remodeling in clonal hematopoiesis and myelodysplasia

Karin D. Prummel[1#], Kevin Woods[2,3#], Maksim Kholmatov[1,4,5#], Eric C. Schmitt[2,3,6], Evi P. Vlachou[1], Mayssa Labyadh[7,8], Rebekka Wehner[9,10,11], Gereon Poschmann[12], Kai Stühler[12,13], Susann Winter[14], Uta Oelschlaegel[14], Manja Wobus[11,14], Logan S. Schwartz[15], Pedro L. Moura[16], Eva Hellström-Lindberg[16], Krishnaraj Rajalingam[17], Matthias Theobald[2,3], Jennifer J. Trowbridge[15], Clémence Carron[7,8,] Thierry Jaffredo[7,8], Marc Schmitz[9,10,11,] Uwe Platzbecker[18], Judith B. Zaugg[1,5*], Borhane Guezguez[2,3,19*]

[1] Molecular Systems Biology Unit, European Molecular Biology Laboratory (EMBL), Heidelberg, Germany. [2] Internal Medicine III., University Medical Center Mainz, Mainz, Germany. [3] German Cancer Consortium (DKTK), Partner Site Frankfurt/Mainz, German Cancer Research Center (DKFZ), Heidelberg, Germany. [4] Laboratory of Systems Biology and Genetics, Institute of Bioengineering, School of Life Sciences, École Polytechnique Fédérale de Lausanne (EPFL), Lausanne, Switzerland. [5] Department of Biomedicine, University Hospital Basel, University of Basel, Basel, Switzerland. [6] Institute of Molecular Biology (IMB), Mainz, Germany. [7] Sorbonne Université, CNRS, INSERM, Development, Adaptation and Ageing, Paris, France. [8] Sorbonne Université, CNRS, Inserm, Institut de Biologie Paris-Seine (IBPS), Paris, France. [9] Institute of Immunology, Faculty of Medicine Carl Gustav Carus, TU Dresden, Dresden, Germany. [10] National Center for Tumor Diseases (NCT), Partner Site Dresden, Germany. [11] German Cancer Consortium (DKTK), Partner Site Dresden, DKFZ, Heidelberg, Germany. [12] Institute of Molecular Medicine I, Proteome Research, University Hospital and Medical Faculty, Heinrich Heine University Düsseldorf, Düsseldorf, Germany. [13] Molecular Proteomics Laboratory, Biological Medical Research Centre (BMFZ), Heinrich Heine University Düsseldorf, Düsseldorf, Germany. [14] Department of Internal Medicine I, Faculty of Medicine Carl Gustav Carus, TU Dresden, Dresden, Germany. [15] The Jackson Laboratory, Bar Harbor, ME, USA. [16] Department of Medicine Huddinge, Center for Hematology and Regenerative Medicine, Karolinska Institute, Huddinge, Sweden. [17] Cell Biology Unit, University Medical Center Mainz, Mainz, Germany. [18] University Hospital Carl Gustav Carus, Dresden, Germany. [19] Research Center for Immunotherapy (FZI), University Medical Centre of the Johannes Gutenberg University Mainz, Mainz, Germany

[#] Equally Contributing Authors. * Corresponding Authors bguezgue@uni-mainz.de and judith.zaugg@unibas.ch

<u>Supplementary Figures 1-12</u>

FIGURE S1

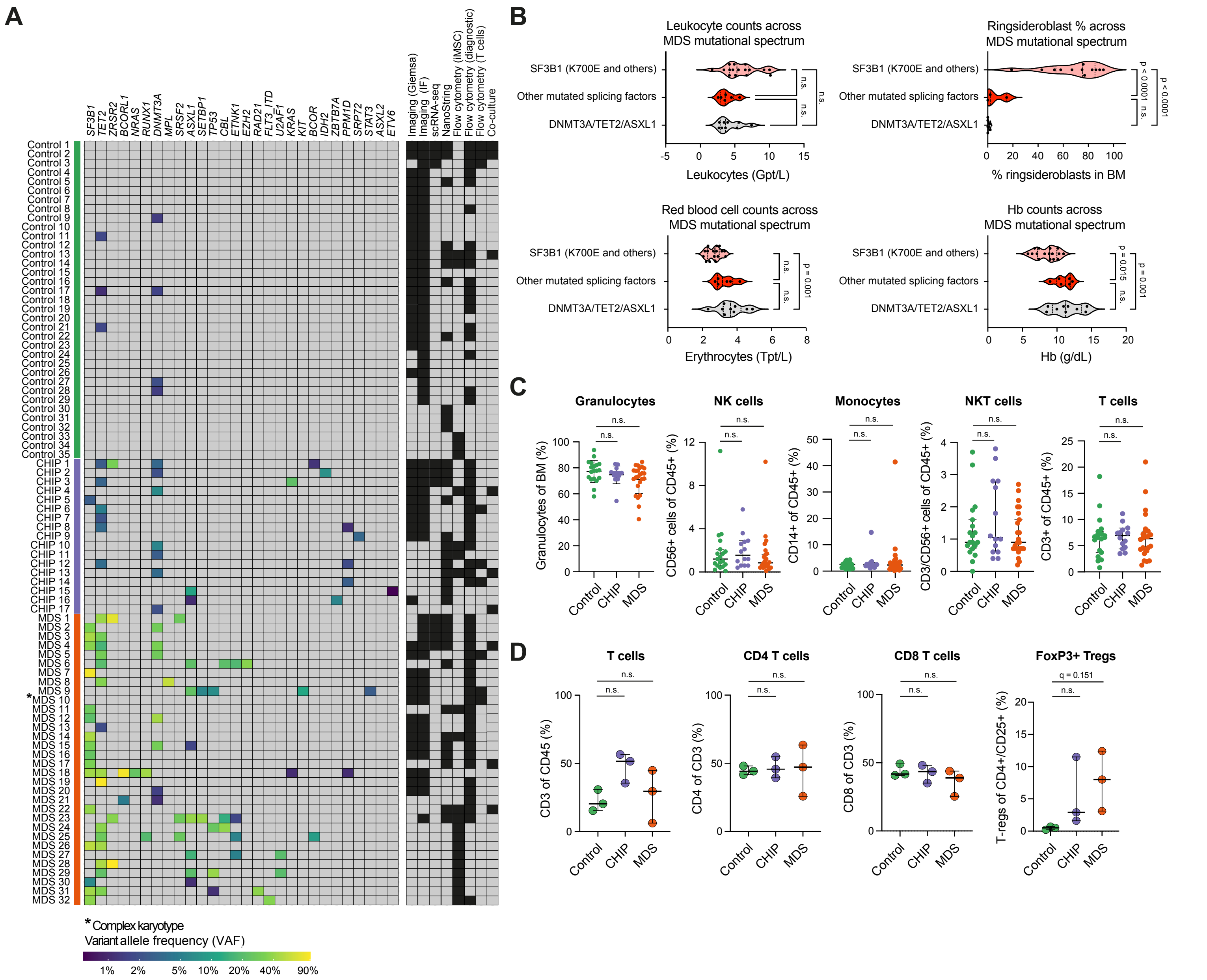

Supplementary Figure 1: Donor information and diagnostic flow cytometry of CHIP/MDS cohort.

(A) Heatmap of variant allele frequencies (VAFs) for detected mutations. For donors with multiple mutations in the same gene, only the highest VAF is shown. * indicates complex karyotype. **(B)** Violin plots representing peripheral blood counts across MDS mutational spectrum of patient's samples of the study (see also **Supplementary Table 1**) (SF3B1 *n*=16, DNMT3A/TET2/ASXL1 *n*=9, other mutated splicing factors *n*=6). Medians with 95% confidence intervals are shown. Statistical test: one-way ANOVA, Tukey's test. **(C)** Quantification of indicated cell type fractions in total BM (granulocytes) and $CD45^+$ BM (rest) from fresh non-Ficoll BM aspirates (RBC lysis only) (Control *n*=18, CHIP *n*=13, MDS *n*=23). Medians with 95% confidence intervals are shown, one-way ANOVA, with FDR correction. **(D)** Frequencies of $CD3^+$, $CD4^+$, $CD8^+$, and regulatory T cells (Tregs, $CD4^+/CD25^+/Foxp3^+$) within $CD45^+$ population of BM aspirates (post-Ficoll) across Control, CHIP, and MDS (*n*=3/condition). Statistical test: one-way ANOVA, Tukey's test. Source data are provided in the Source Data file. n.s. = not significant.

FIGURE S2

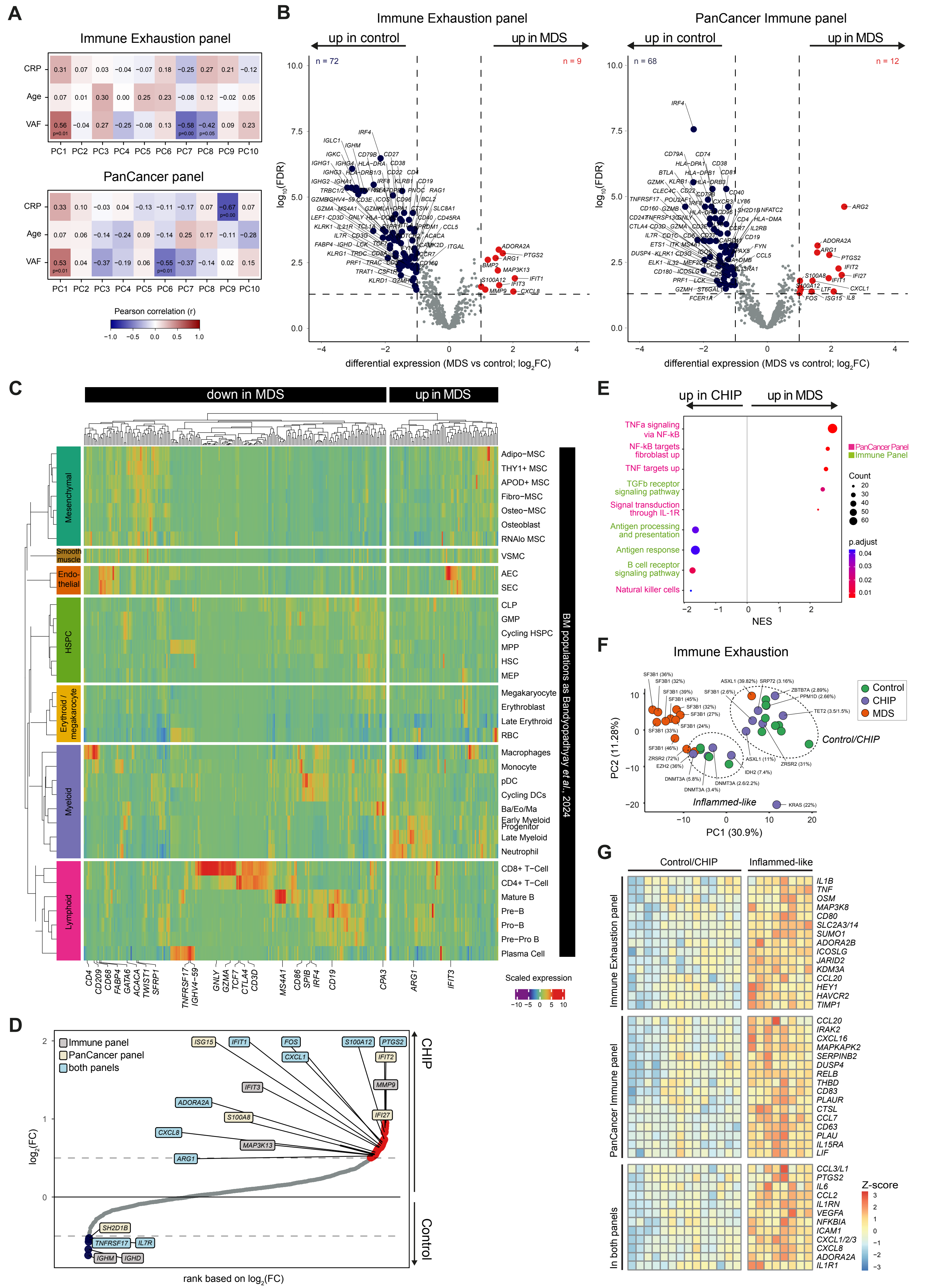
A
Immune Exhaustion panel
PanCancer panel
Pearson correlation (r)
B
Immune Exhaustion panel
PanCancer Immune panel
up in control
up in MDS
differential expression (MDS vs control; log2FC)
C
down in MDS
up in MDS
BM populations as Bandyopadhyay et al., 2024
Scaled expression
D
Immune panel
PanCancer panel
both panels
CHIP
Control
rank based on log2(FC)
E
up in CHIP
up in MDS
NES
F
Immune Exhaustion
Control/CHIP
Inflammed-like
PC1 (30.9%)
PC2 (11.28%)
G
Control/CHIP
Inflammed-like
Immune Exhaustion panel
PanCancer Immune panel
In both panels
Z-score

Supplementary Figure 2: Transcriptional profiling of CHIP and MDS cohort using NanoString immune gene panels.

(A) Correlation heatmaps showing associations of the Top 10 principle components (PC1-10) from the NanoString Immune Exhaustion and PanCancer Immune panels with metadata: age, VAF, and CRP (C-reactive protein). Statistical test: Pearson's correlation coefficient and two-sided unpaired Student's *t*-test. **(B)** Volcano plots of differentially expressed genes in MDS vs. Control for both panels: Immune Exhaustion (72 downregulated, 9 upregulated) and PanCancer Immune (68 downregulated, 12 upregulated). Differential expression was tested using linear models and empirical Bayes moderation as implemented in the limma package. Statistical significance: *p*-values were determined using Benjamini–Hochberg FDR correction. **(C)** Heatmap displaying the expression levels of MDS versus Control differentially expressed genes across cell types in the healthy BM scRNA-seq [47]. Gene expression is averaged and scaled within each cell type, using the authors' original cell type annotations. **(D)** Rank plot showing $\log_2$ fold changes between CHIP and Control samples across both NanoString panels (color-coded boxes). Genes with $|\log_2 FC| > 0.5$ are labeled with blue (down) and red (up) dots in gene expression between MDS and control samples (D) and CHIP versus control samples. Genes overlapping with the significant differential genes between Control and MDS (B) are specifically highlighted. **(E)** GSEA for genes up- and downregulated in MDS versus CHIP across both NanoString panels. Normalized enrichment scores (NES) are shown for significantly enriched pathways, with dot color indicating adjusted *p*-value and size indicating gene count. Terms are color-coded by panel: Immune Exhaustion panel (green), PanCancer Immune panel (pink). **(F)** PCA of the Immune Exhaustion panel, highlighting the variant allele frequencies (VAF) of the CHIP and MDS donors (**Supplementary Fig. 1A**). The CHIP and Control donors can be grouped into Inflammed-like and Control/CHIP groups. **(G)** Extended heatmap of differentially expressed genes from both NanoString panels between CHIP (Inflammed-like) and Control (Control/CHIP) groups. Genes are grouped by panel of origin and expression values are scaled by gene (Z-score). Source data are provided in the Source Data file.

FIGURE S3

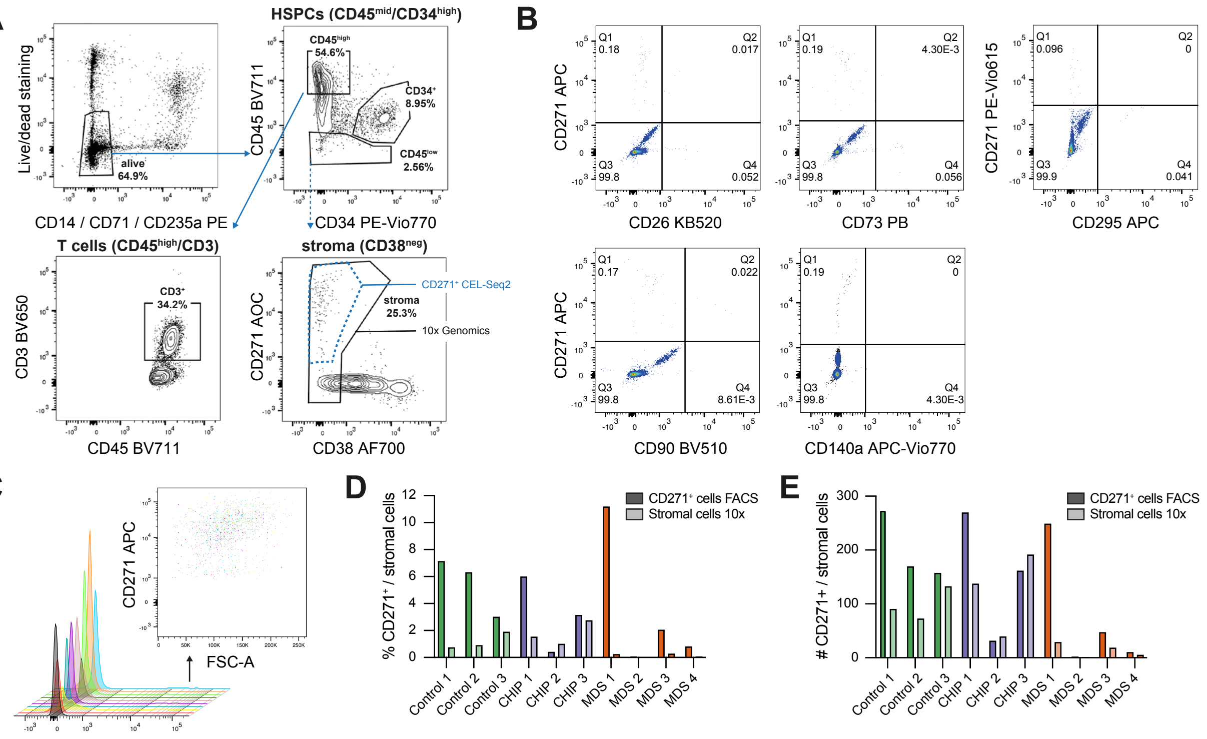

Supplementary Figure 3: FACS strategy for scRNA-seq data sets

(A) Flow cytometry gating strategy for human BM aspirates for 10x Genomics and CEL-Seq2 scRNA-seq experiments. Schematic is showing the gating used to isolate HSPCs ($CD45^{mid}CD34^{+}$), T cells ($CD45^{+}CD3^{+}$), and stromal populations (10x Genomics: $CD45^{-}CD14^{-}CD71^{-}CD235a^{-}CD38^{-}$, CEL-seq2: $CD271^{+}$). **(B)** Surface marker analysis of stromal cells: flow cytometry dot plots displaying $CD271^{+}$ expression together with additional stromal surface markers (CD26, CD73, CD295, CD90, and CD140a). CD271 marks the majority of the stromal cells compared to the other markers tested. **(C)** Flow cytometry plot of CD271⁺ (MSCs) plotted against the forward scatter (FSC-A) after gating out the lineage-positive cells. (D-E) Quantification of $CD271^{+}$ stromal cells across all donors included in scRNA-seq experiments. **(D)** Percentage and **(E)** absolute numbers of $CD271^{+}$ cells in BM aspirates by FACS (dark bars) compared to the respective recovery of stromal cells in the scRNA-seq dataset (light bars) for each donor. Source data are provided in the Source Data file.

FIGURE S4

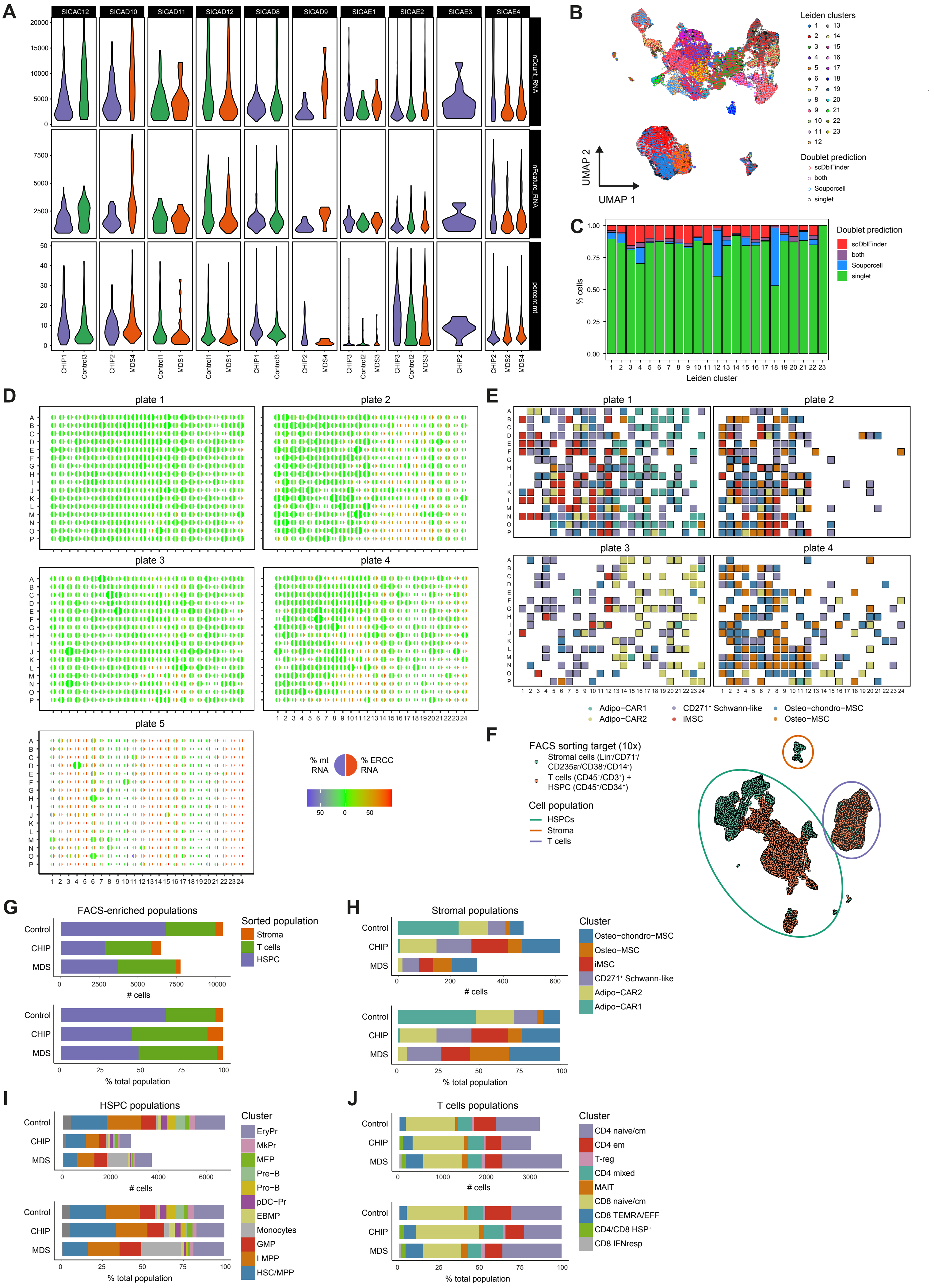

A
SIGAC12
SIGAD10
SIGAD11
SIGAD12
SIGAD8
SIGAD9
SIGAE1
SIGAE2
SIGAE3
SIGAE4
nCount_RNA
nFeature_RNA
percent.mt
B
Leiden clusters
Doublet prediction
scDblFinder
both
Souporcell
singlet
UMAP 1
UMAP 2
C
% cells
Leiden cluster
D
plate 1
plate 2
plate 3
plate 4
plate 5
% mt RNA
% ERCC RNA
E
Adipo−CAR1
Adipo−CAR2
CD271⁺ Schwann-like
iMSC
Osteo−chondro−MSC
Osteo−MSC
F
FACS sorting target (10x)
Stromal cells (Lin⁻/CD71⁻/CD235a⁻/CD38⁻/CD14⁻)
T cells (CD45⁺/CD3⁺) + HSPC (CD45⁺/CD34⁺)
Cell population
HSPCs
Stroma
T cells
G
FACS-enriched populations
Sorted population
Stroma
T cells
HSPC
Control
CHIP
MDS
cells
% total population
H
Stromal populations
Cluster
Osteo−chondro−MSC
Osteo−MSC
iMSC
CD271⁺ Schwann-like
Adipo−CAR2
Adipo−CAR1
I
HSPC populations
Cluster
EryPr
MkPr
MEP
Pre−B
Pro−B
pDC−Pr
EBMP
Monocytes
GMP
LMPP
HSC/MPP
J
T cells populations
Cluster
CD4 naive/cm
CD4 em
T−reg
CD4 mixed
MAIT
CD8 naive/cm
CD8 TEMRA/EFF
CD4/CD8 HSP⁺
CD8 IFNresp

Supplementary Figure 4: Quality control of scRNA-seq data sets

(A) Violin plots showing the distribution of total RNA counts (nCount_RNA = UMIs), detected genes per cell (nFeature_RNA = unique genes), and mitochondrial RNA content (percent.mt) across tscRNA-seq libraries and different donors. **(B)** UMAP visualization of the unfiltered dataset, displaying Leiden clustering alongside predicted singlets and doublets identified with scDbFinder and Souporcell (color-based). **(C)** Bar plot quantifying singlet/doublet proportion per Leiden cluster from (B). Clusters 12 and 18, enriched for doublets, were excluded from downstream analysis. **(D)** Quality control metrics for CEL-Seq2 plates (1-5). Dot plots show mitochondrial RNA content (% mtRNA, blue) and ERCC RNA spike-in proportion (% ERCC, red), reflecting RNA integrity and technical noise. **(E)** Cell identity assignment across CEL-Seq2 plates (1-4), showing stromal subtype distribution per well. **(F)** UMAP representation of the unfiltered 10x scRNA-seq dataset, highlighting the FACS-based enrichment strategy prior to library generation. Cells are colored by sorted population. contamination of stromal libraries with HSPCs, particularly erythroid and early monocyte progenitors from MDS samples, is indicated. **(G-J)** Bar plots summarizing cell type composition across conditions. Within (G) the distribution of the major FACS-enriched compartments; (H) stromal subtypes; (I) HSPC subclusters; and (J) T cell subsets. For each panel, top bars represent absolute cell counts and bottom bars show relative proportions. Source data are provided in the Source Data file and Supplementary Data 7.

FIGURE S5

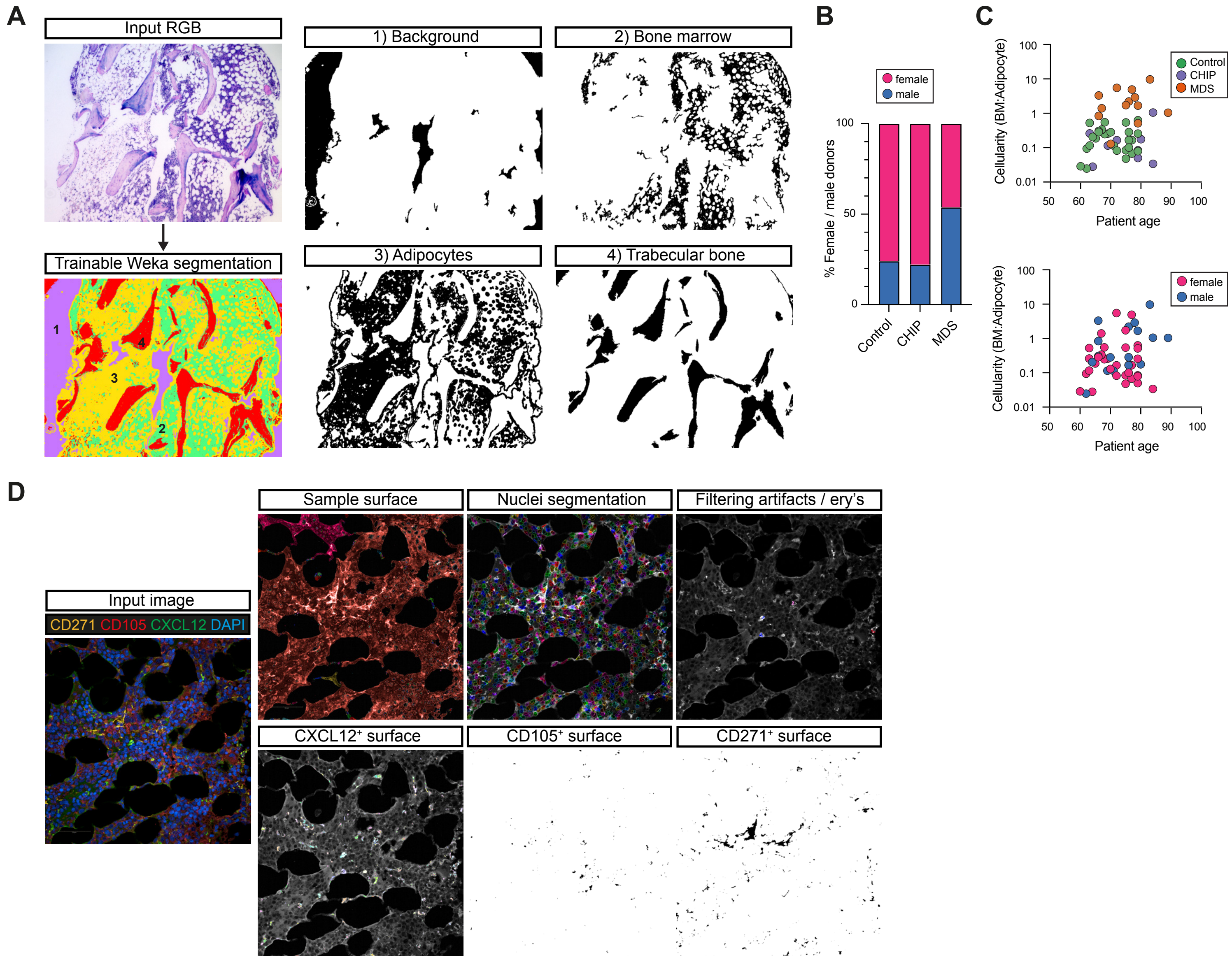

Supplementary Figure 5: Segmentation and quantification of Giemsa and immunofluorescence imaging of bone biopsies

(A) Image sequence analysis of cellularity of Giemsa-stained BM sections. Trainable Weka-segmentation in Fiji was applied to classify RGB images into four classes: 1) background (purple), 2) bone marrow (green), 3) adipocytes (yellow), and 4) trabecular bone (red). After filtering and cleanup, masks were generated and used for cellularity analysis in **Fig. 3C** (surface area bone marrow/surface area adipocytes). **(B)** Proportion of male (blue) and female (magenta) included in Giemsa staining and cellularity quantification. **(C)** Scatter plots showing the relationship between cellularity, age, condition, and sex. **(D)** Semi-quantitative analysis of BM FFPE trephine samples using PerkinElmer Harmony software. quality control excluded fields with artifacts or uneven staining. For each sample, 50-300 fields were analyzed using a standardized pipeline for automated image analysis. Automatic thresholds were used to detect positive (surface) staining, while nuclei segmentation was optimized by applying a Gaussian blur to DAPI staining in selected samples to avoid over-segmentation. Additional filtering removed small artifacts and autofluorescent background signals, primarily from erythrocytes (ery's). For all populations of interest, the overlap (%) and neighbor distance metrics were calculated. Source data are provided in the Source Data file.

FIGURE S6

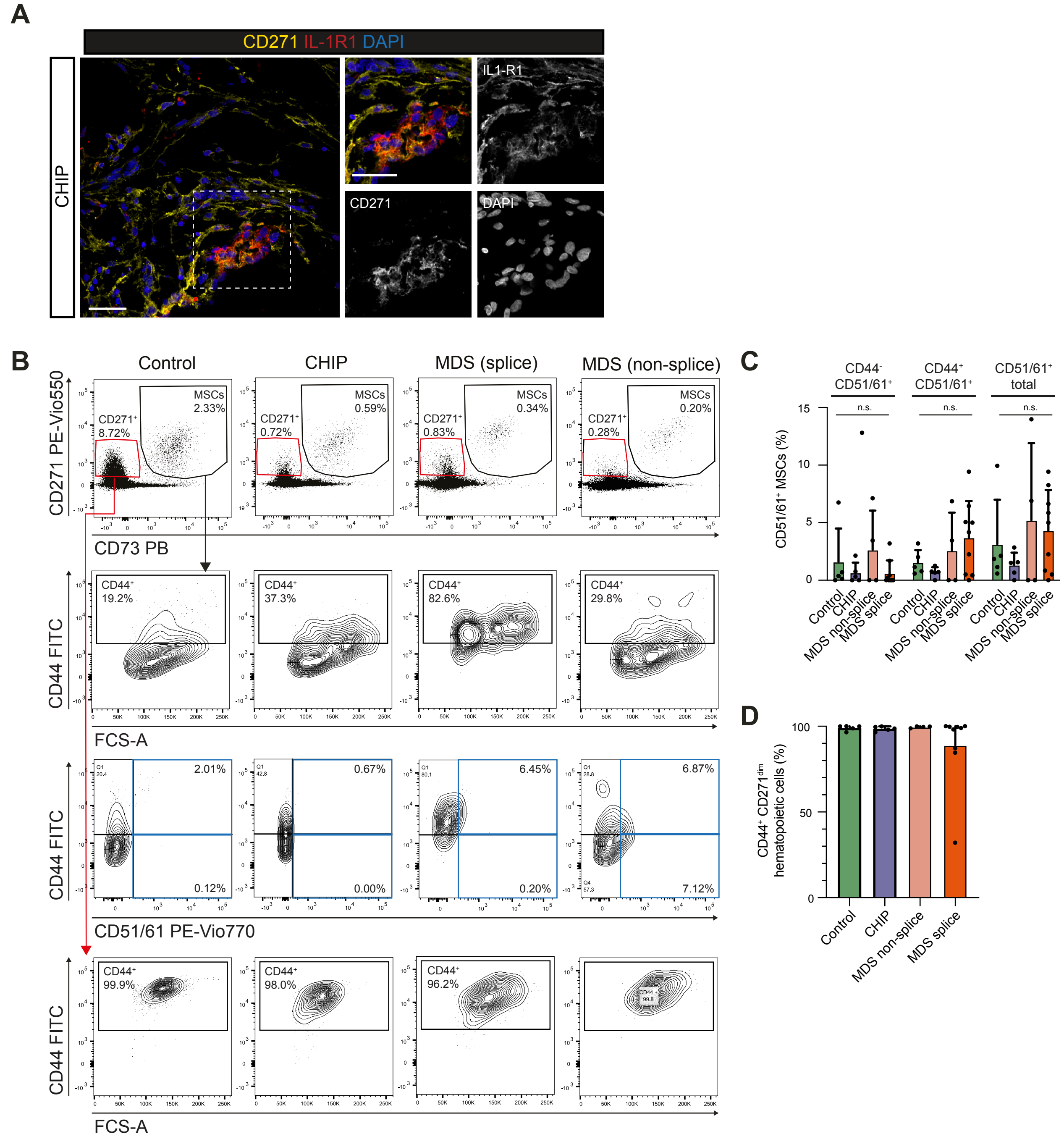

Supplementary Figure 6: Characterization strategy of iMSC by flow cytometry and tissue imaging

(A) Immunofluorescence staining of BM sections from a CHIP donor, showing co-staining of IL-1R1 (red) and CD271 (yellow). Insets highlight representative areas containing inflammatory IL-1R1⁺CD271⁺ stromal cells. Nuclei are counterstained with DAPI (blue). Scale bars: 25 µm. **(B)** Representative flow cytometry dot plots from Control, CHIP, and MDS (splice and non-splice) donors showing: CD271⁺/CD73⁺ stromal population (top row, used for the analysis in **Fig. 3H,I**); CD44⁺ MSCs (second row); CD44⁺/CD51/61⁺ double-positive MSCs (third row). The bottom row shows the CD44 expression in the CD271^{dim} population with low FCS-A (gated in red). **(C)** Quantification of the CD44⁺ and CD51/61⁺ MSC subsets as percentage of total MSCs in Control (*n*=5), CHIP (*n*=5), and MDS (*n*=13) samples from (B). Means with SD are shown, two-way ANOVA with FDR correction. **(D)** Percentage of CD44⁺CD271^{dim} cells across donor groups. Source data are provided in the Source Data file. n.s. = not significant.

FIGURE S7

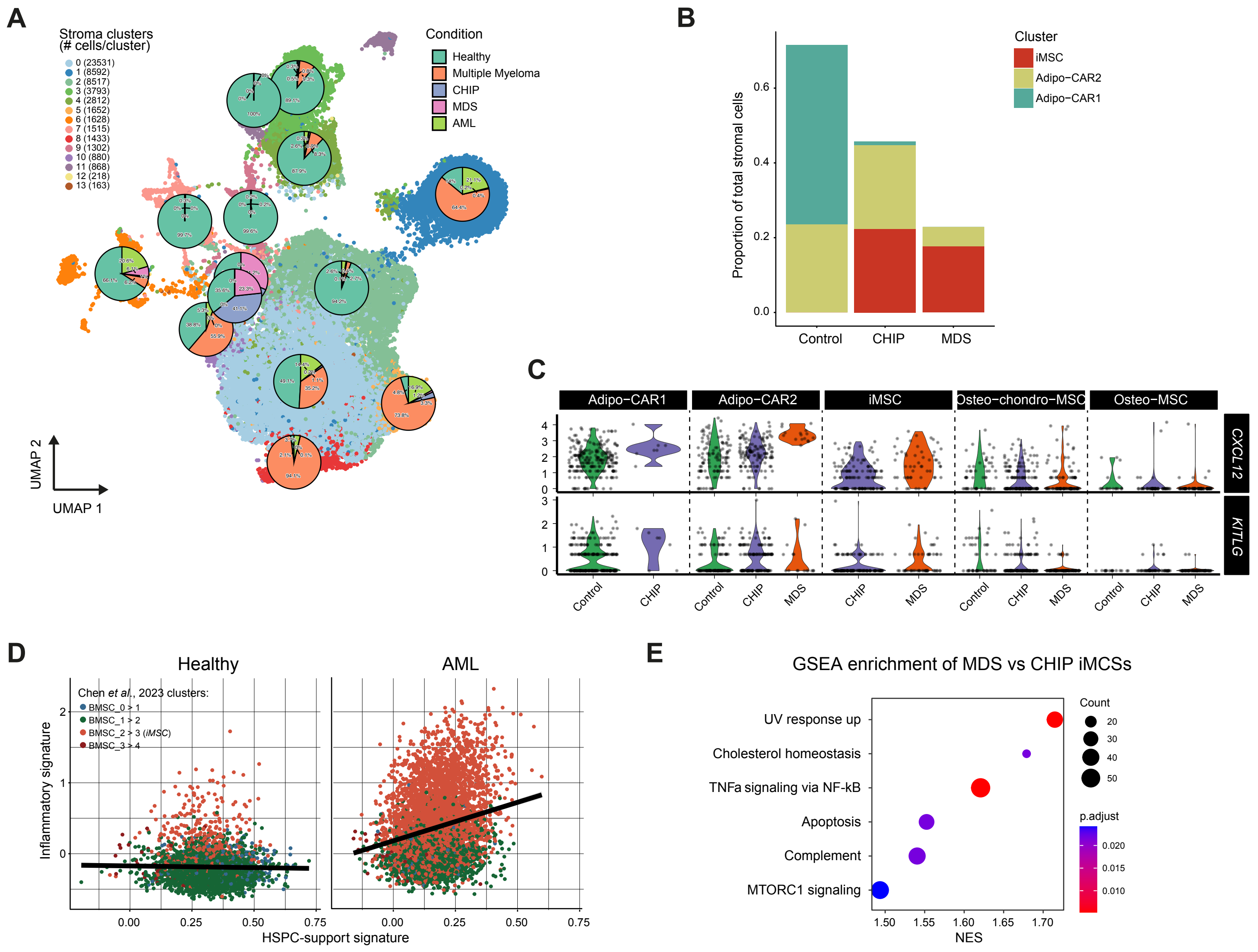

Supplementary Figure 7: Inflammatory signatures in stromal cell clusters across Control, CHIP, MDS, MM, and AML

(A) UMAP of stromal cell populations from the integrated datasets (see Fig. 3), showing 14 distinct stromal clusters. Each cluster is annotated by a pie chart indicating the relative contribution of each condition (healthy, MM, CHIP, MDS, AML). Cluster sizes (cell numbers) are indicated. **(B)** Bar plot visualizing the proportion of Adipo-CAR1/2 and iMSCs among all stromal cells across the different conditions (Control, CHIP, MDS). **(C)** Normalized expression of CXCL12 and KITLG in stromal clusters across the conditions. **(D)** Scatter plots showing the relationship between our curated HSPC-support and inflammatory gene signature scores in stromal cells of healthy and AML BM[83]. Each dot represents a single cell, colored by the stromal subtype from the original study. Black trend lines indicate the direction of the correlation per cndition. **(E)** GSEA comparing iMSCs from MDS versus CHIP samples using Hallmark gene sets (MSigDB). Dot plot shows normalized enrichment scores (NES), adjusted *p*-values (color scale), and gene set size (dot size). Source data are provided in the Source Data file and Supplementary Data 7.

FIGURE S8

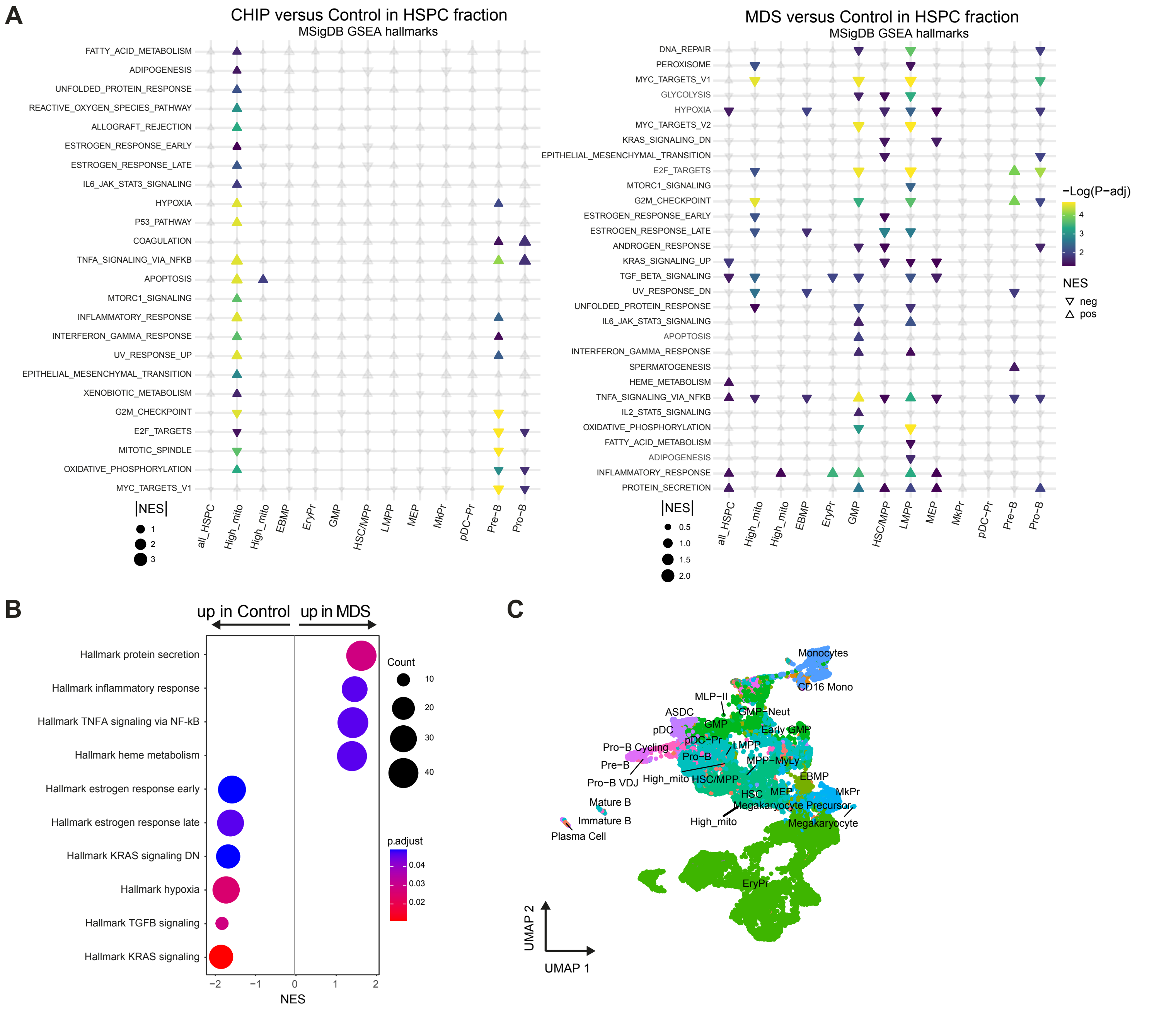

Supplementary Figure 8: Gene expression differences in HSPCs across Control, CHIP, and SF3B1^{WT}/SF3B1^{MUT} MDS

(A) GSEA using Hallmark gene sets (MSigDB) comparing CHIP versus Control (left) or MDS versus Control (right) across HSPC cell types **(Methods)**. Each triangle represents a specific hallmark within an annotated HSPC population. Color indicates adjusted *p*-values (with grey not significant), triangle direction indicates direction of enrichment (up- or downregulated), and size represents normalized enrichment score (NES). **(B)** GSEA results for all HSPC populations (excluding monocytes), comparing MDS and Control samples. Color scale indicates the adjusted *p*-value and dot size the gene set size. **(C)** UMAP of the HSPC-enriched cell fraction prior removal of contaminating cells from the stromal FACS-enrichment strategy. Manually annotated cell types were integrated with labels transferred from BoneMarrowMap[60]. All populations corresponding to distinct stages of erythropoiesis were merged to perform differential expression analysis between *SF3B1^{MUT}* and *SF3B1^{WT}* predictions in MDS samples using SpliceUp **(Fig. 5D-F)**. Source data are provided in the Source Data file.

FIGURE S9

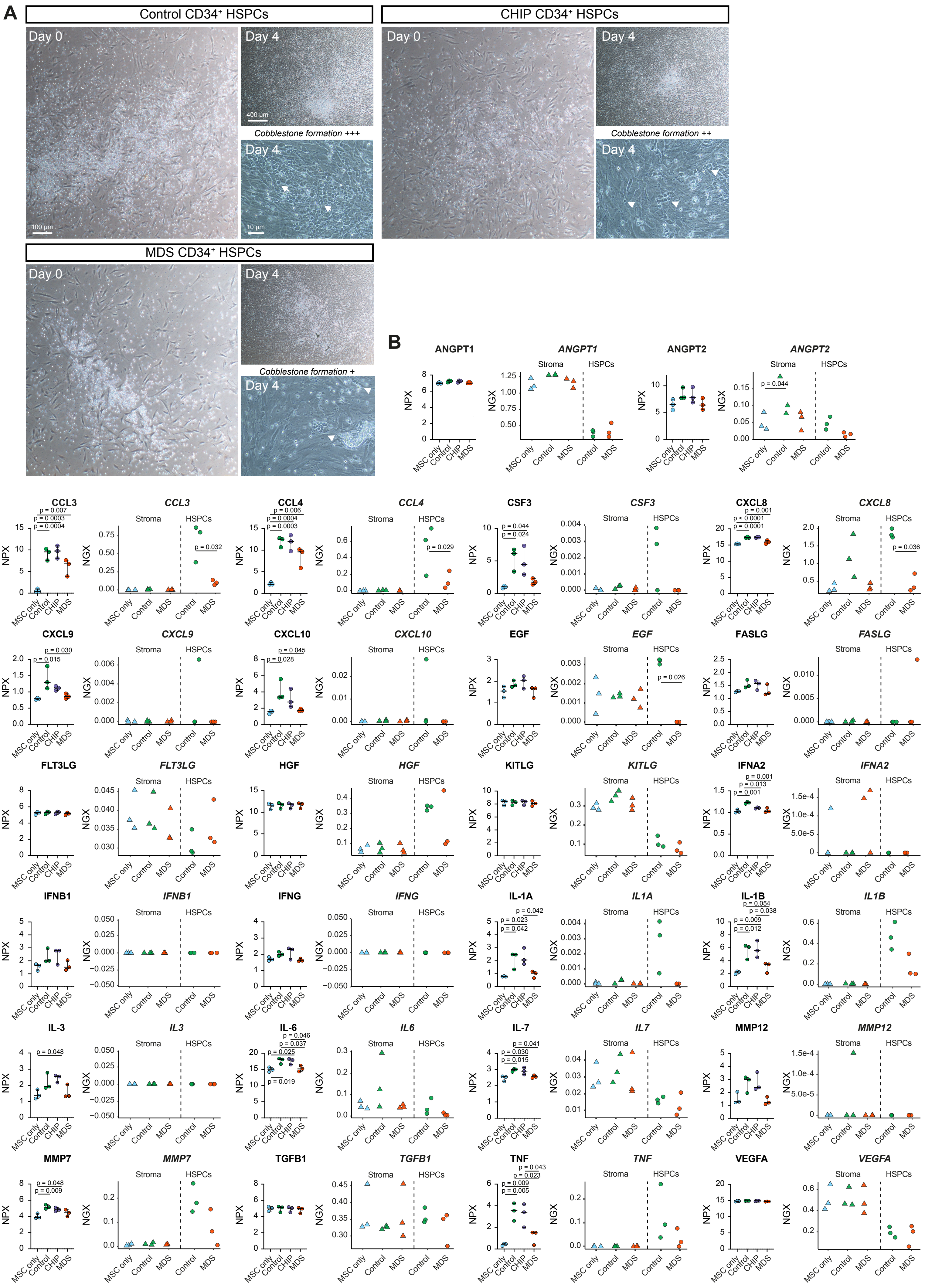

A
Control CD34⁺ HSPCs
Day 0
Day 4
400 µm
100 µm
10 µm
Cobblestone formation +++
CHIP CD34⁺ HSPCs
Cobblestone formation ++
MDS CD34⁺ HSPCs
Cobblestone formation +
B
ANGPT1
ANGPT2
CCL3
CCL4
CSF3
CXCL8
CXCL9
CXCL10
EGF
FASLG
FLT3LG
HGF
KITLG
IFNA2
IFNB1
IFNG
IL-1A
IL1A
IL-1B
IL1B
IL-3
IL3
IL-6
IL6
IL-7
IL7
MMP12
MMP7
TGFB1
TNF
VEGFA
NPX
NGX
Stroma
HSPCs
MSC only
Control
CHIP
MDS

Supplementary Figure 9: MSC–HSPC co-cultures with cytokine profiling and scRNA-seq in Control, CHIP, MDS

(A) Representative microscopy images from co-culture experiments using primary $CD34^+$ HSPCs from Control, CHIP, or MDS donors and primary BM-derived MSCs (*n*=3/conditions). Images show day 0 (start of co-culture) and day 4 (endpoint). Insets show higher magnification of cobblestone areas (arrows), a proxy for stromal-HSPC interaction and HSPC-stemness (HSCs/MPPs). Qualitative cobblestone scoring (+++, ++, +) is based on density and clustering of phase-dark hematopoietic cells under the MSC layer. Scale bars annotated in the panels. **(B)** Secreted cytokines and gene expression profiling of stromal and HSPC compartments post co-culture. Left dot plot: mean normalized protein expression (NPX) values (3 technical of 3 biological replicates) from Olink across 26 cytokines/growth factors in the culture supernatant across conditions (MSC mono-culture (MSC-only), Control, CHIP, MDS, *n*=3/condition). Right dot plot: corresponding normalized gene expression (NGX) values for the secreted factors from scRNA-seq of the total recovered stromal and HSPC fractions from MSC mono-culture (MSC-only), and Control and MDS co-cultures. Medians with 95% confidence intervals are shown. Statistical tests: one-way ANOVA, Tukey's test (Olink) and two-sided unpaired Student's *t*-test (scRNA-seq). Source data are provided in the Source Data file. n.s. = not significant.

FIGURE S10

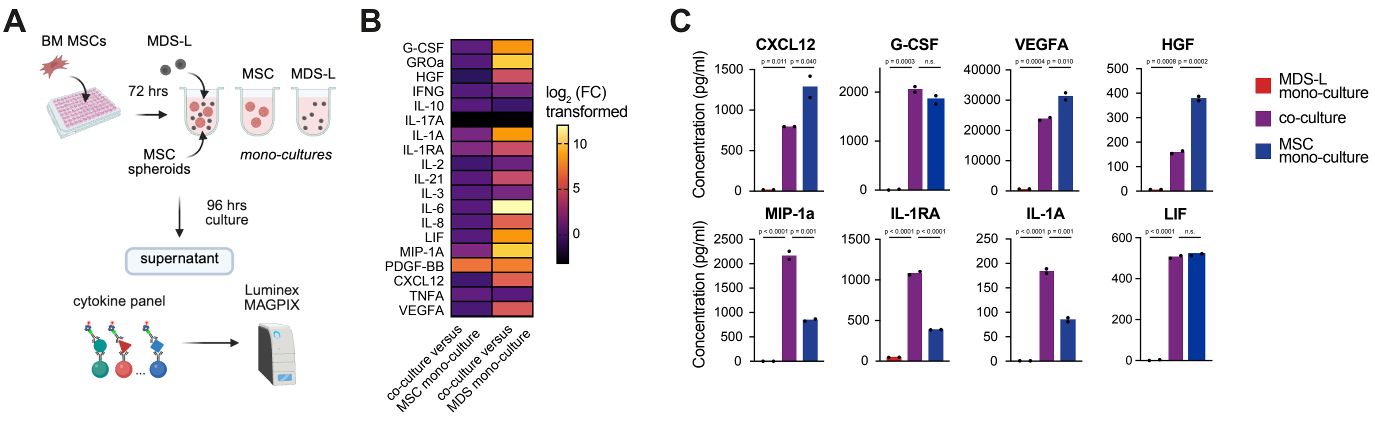

Supplementary Figure 10: Luminex-based cytokine profiling of BM-MSC and MDS-L co-cultures.

(A) Schematic representation of the experimental setup. Immortalized BM-derived MSCs were pre-cultured for 72 hrs to form spheroids, then co-cultured with MDS-L cells for 96 hrs. Parallel mono-cultures of MSCs and MDS-L cells were included as controls. Supernatants were collected and analyzed using Luminex. Created in BioRender. Prummel, K. (2025) https://BioRender.com/cueksux. **(B)** Heatmap summarizing cytokine abundance across three comparisons: co-culture versus MSC mono-culture and co-culture versus MDS-L mono-culture (n=2/condition). Log_2 fold change values are color-scaled. **(C)** Bar plots showing concentrations (pg/ml) of selected cytokines across conditions. Co-culture (striped bars) led to reduced levels of multiple MSC-derived factors (e.g., CXCL12, MIP-1α, IL-1RA, IL1α) compared to MSC mono-culture (blue), while G-CSF remained unchanged and LIF was not significantly altered. MDS-L mono-culture levels (red) are shown for comparison. Statistical test: one-way ANOVA, Tukey's test. Source data are provided in the Source Data file. n.s. = not significant.

FIGURE S11

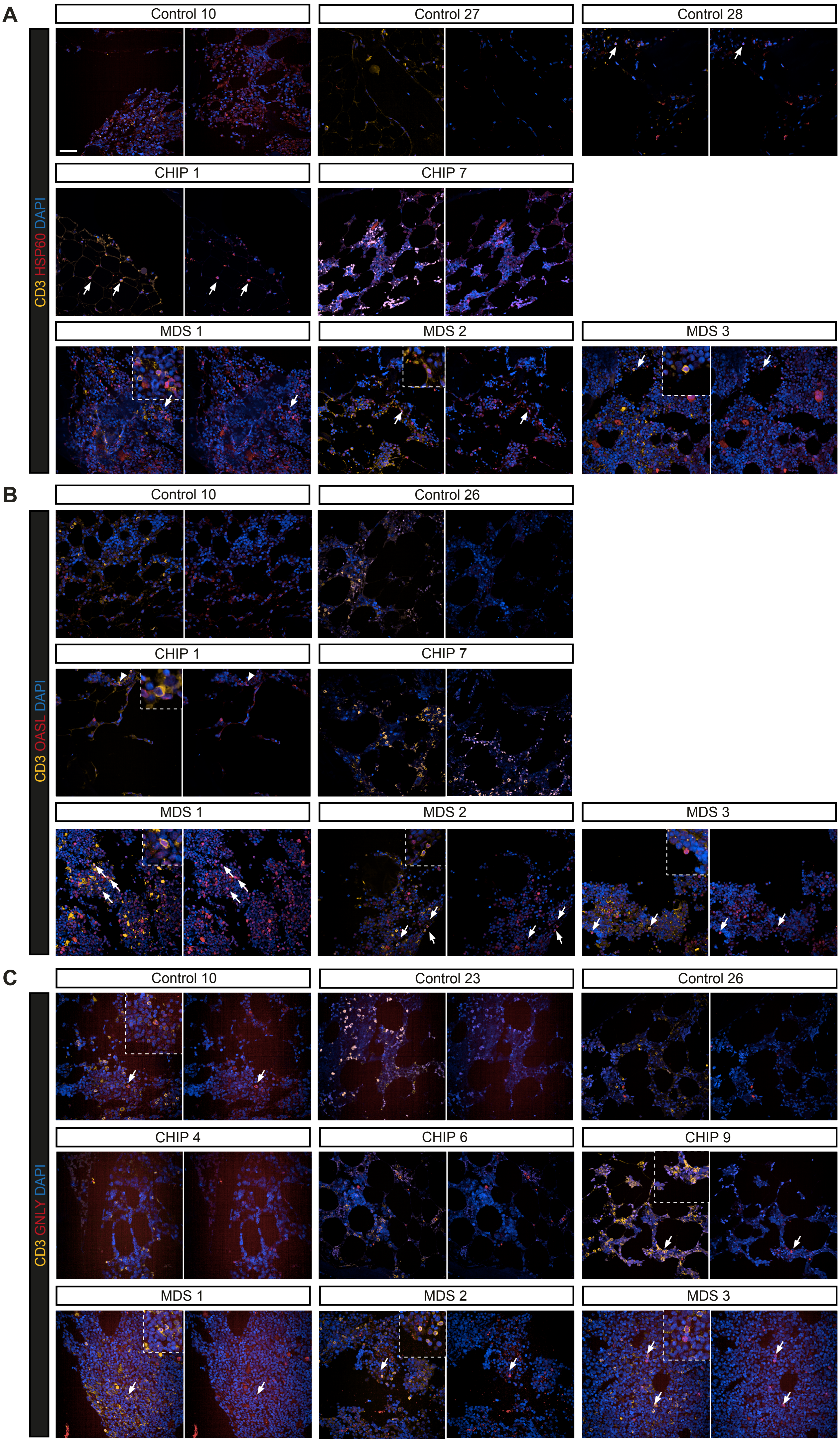

A
Control 10
Control 27
Control 28
CHIP 1
CHIP 7
MDS 1
MDS 2
MDS 3
CD3 HSP60 DAPI
B
Control 10
Control 26
CHIP 1
CHIP 7
MDS 1
MDS 2
MDS 3
CD3 OASL DAPI
C
Control 10
Control 23
Control 26
CHIP 4
CHIP 6
CHIP 9
MDS 1
MDS 2
MDS 3
CD3 GNLY DAPI

Supplementary Figure 11: Visualization of HSP$^+$, IFN-responsive, and T_{EMRA} cytotoxic T cells in the BM of CHIP and MDS

(A-C) Representative images of CD3 T cells (yellow), co-stained with the cell cluster markers HSP60 (red, A), OASL (red, B), or GNLY (red, C). Zoomed-in regions highlights and arrows indicate double-positive cells. Scale bar: 25 µm.

FIGURE S12

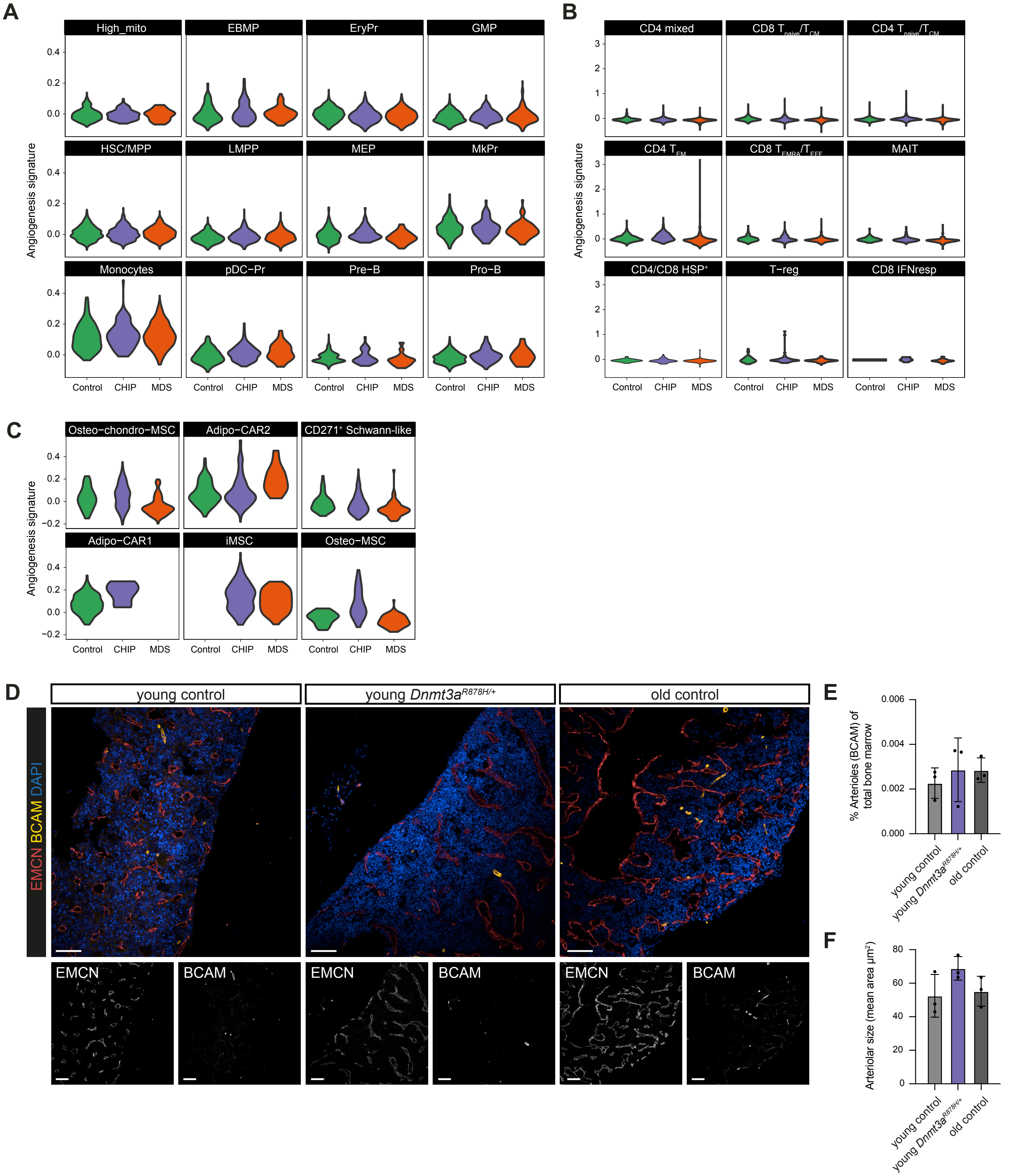

Supplementary Figure 12: Angiogenesis scores across all BM stromal, HSPCs, and T cell clusters and vascular remodeling characterization in CHIP

(A-C) Violin plots representing the angiogenesis signatures in all individual A) HSPC clusters, B) T cell clusters, and C) stromal clusters across control, CHIP, and MDS samples from the scRNA-seq data. **(D)** Representative images of mouse bone section co-stained with sinusoidal (EMCN) and arteriolar (BCAM) markers of young control, young *Dnmt3a*^{R878H}, and old control mice. DAPI (blue) stains the nuclei. Scale bar: 100 µm. **(E)** Quantification of the total arteries/arteriolar (BCAM) area in the mouse femur (*n*=3/condition). Statistical test: two-way ANOVA, Tukey's test. **(F)** Quantification of the mean area of the arteries/arteriolar vasculature (arteries/arteriolar size) (*n*=3/condition). Statistical test: one-way ANOVA with FDR correction. Source data are provided in the Source Data file. n.s. = not significant.
